# Delirium risk and mortality in people with pre-existing severe mental illness: a retrospective cohort study using linked datasets in England

**DOI:** 10.1017/S0033291724002484

**Published:** 2024-10

**Authors:** Yehudit Bauernfreund, Naomi Launders, Graziella Favarato, Joseph F Hayes, David Osborn, Elizabeth L Sampson

**Affiliations:** 1 Division of Psychiatry, University College London, London W1T 7BN, UK; 2 Camden & Islington NHS Foundation Trust, London NW10PE, UK; 3 Department of Psychological Medicine, East London NHS Foundation Trust, Royal London Hospital, London E1 1BU, UK

**Keywords:** bipolar disorder, databases, delirium, psychotic disorders, Schizophrenia

## Abstract

**Background:**

Delirium is a severe neuropsychiatric syndrome caused by physical illness, associated with high mortality. Understanding risk factors for delirium is key to targeting prevention and screening. Whether severe mental illness (SMI) predisposes people to delirium is not known. We aimed to establish whether pre-existing SMI diagnosis is associated with higher risk of delirium diagnosis and mortality following delirium diagnosis.

**Methods:**

A retrospective cohort and nested case–control study using linked primary and secondary healthcare databases from 2000–2017. We identified people diagnosed with SMI, matched to non-SMI comparators. We compared incidence of delirium diagnoses between people with SMI diagnoses and comparators, and between SMI subtypes; schizophrenia, bipolar disorder and ‘other psychosis’. We compared 30-day mortality following a hospitalisation involving delirium between people with SMI diagnoses and comparators, and between SMI subtypes.

**Results:**

We identified 20 566 people with SMI diagnoses, matched to 71 374 comparators. Risk of delirium diagnosis was higher for all SMI subtypes, with a higher risk conferred by SMI in the under 65-year group, (aHR:7.65, 95% CI 5.45–10.7, ⩾65-year group: aHR:3.35, 95% CI 2.77–4.05). Compared to people without SMI, people with an SMI diagnosis overall had no difference in 30-day mortality following a hospitalisation involving delirium (OR:0.66, 95% CI 0.38–1.14).

**Conclusions:**

We found an association between SMI and delirium diagnoses. People with SMI may be more vulnerable to delirium when in hospital than people without SMI. There are limitations to using electronic healthcare records and further prospective study is needed to confirm these findings.

## Introduction

Delirium is a severe neuropsychiatric syndrome caused by physical illness and acute cerebral stress (American Psychiatric Association, 2013). It is common, affecting up to 32% of hospitalised patients (Koirala et al., 2020). It is associated with poor outcomes including increased mortality and risk of future dementia (Richardson et al., 2020; Salluh et al., 2015), and is costly for healthcare providers (Kinchin, Mitchell, Agar, & Trépel, 2021). Despite its high prevalence and poor prognosis, delirium remains under-diagnosed and treated (Ritter et al., 2018). There has been an increase in reported delirium incidence since 2010 (Ibitoye, Jackson, Davis, & MacLullich, 2023a) which coincides with publication of NICE delirium guidelines and the ‘Think Delirium’ campaign (NICE, 2010; Young, 2010).

Delirium is understood as ‘acute brain failure’ (Maldonado, 2018), with decompensation more likely if the brain is under chronic stress. Organic brain diseases such as dementia and stroke are predisposing risk factors for delirium (Wilson et al., 2020). Less is known as to whether severe mental illnesses (SMI), which also cause chronic stress (Abé et al., 2022; Andreasen et al., 2011), increase delirium risk. SMI, including schizophrenia, bipolar disorder and other psychoses (N. H. S. Digital, 2020/21) affects approximately half a million adults in England (Public Health England, 2018). People with SMI may be at higher risk of delirium due to high rates of physical morbidity, psychotropic medications and frailty (Launders, Hayes, Price, Marston, & Osborn, 2022a; Launders, Hayes, Price, & Osborn, 2022b; Osborn et al., 2008; Pearson et al., 2022), all of which predispose to delirium (Ahmed, Leurent, & Sampson, 2014). People with SMI may also have structural and functional neurological vulnerability to delirium through network disintegration and blood brain barrier disruption (Pollak et al., 2018; Skåtun et al., 2016).

Despite this theoretical vulnerability, there is limited evidence as to whether SMI increases risk of delirium. Recorded diagnoses of delirium in SMI have increased in recent years (Bauernfreund et al., 2022), but how this compares to rates within the general population is not known. Risk of delirium increases markedly for each year of age after 65 (Pandharipande et al., 2006), but how relative risk of delirium for people with SMI differs by age group is not known. Furthermore, delirium may be harder to identify in people with SMI, due to an overlap in symptoms, stigma and diagnostic overshadowing (Fiorillo & Sartorius, 2021; Kishi et al., 2007).

The mortality of delirium in people with SMI has not been previously studied. Delirium during hospitalisation is associated with increased risk of mortality in the general population (Witlox et al., 2010), likely due to both the delirium itself and the underlying illness, although the pathophysiology is not well understood (Bellelli et al., 2007). People with SMI have higher mortality for cardiovascular disease and cancer (Correll et al., 2017; Launders, Scolamiero, Osborn, & Hayes, 2022c). Understanding whether higher mortality occurs with delirium will address the importance of delirium preventative measures in this group.

Hypotheses:

1. SMI diagnosis will be associated with higher incidence of delirium diagnosis, in both younger (18–64 years) and older (⩾65 years) adults.
2. SMI diagnosis will be associated with higher odds of 30-day mortality following a hospitalisation involving delirium.

## Methods

### Study design

We used a retrospective matched cohort study using anonymised linked data collected from electronic health records (EHRs), according to methods previously described (Launders et al., 2022a), to establish incidence of delirium diagnoses based on SMI status. Secondly, we conducted an unmatched nested case–control study on the sub-group who received a delirium diagnosis to analyze mortality following delirium by SMI status. This study is reported according to STROBE guidelines (von Elm et al., 2007).

### Data sources

The Clinical Practice Research Datalink (CPRD) Gold and Aurum databases hold anonymized routinely collected patient records from 60 million patients across 2000 UK primary care practices (National Institute for Health & Care Research, 2022), broadly demographically representative of the UK population (Herrett et al., 2015). Diagnoses for SMI have been previously established in the database (Nazareth, King, Haines, Rangel, & Myers, 1993) and are likely well-recorded due to NHS Quality Outcomes Framework incentivisation (N. H. S. Digital, 2020/21).

Data linkage to other databases is available for a proportion of CPRD patients registered with a GP practice in England who have consented to participate in the NHS England linkage scheme (CPRD, 2024b). This is done through matching of NHS number, date of birth, sex and postcode; thus errors or missing data in these variables may prevent linkage. We linked data from CPRD to Hospital Episode Statistics Admitted Patient Care (HES-APC) to capture recorded delirium diagnoses in secondary care. HES-APC stores data on all NHS-funded hospitalizations to general and psychiatric hospitals in England, and for this study, data was available from 1 April 2000 to 31 March 2017. We excluded maternity and regular repeat hospitalizations as classified in HES (i.e. for renal dialysis or cancer treatment). We also linked data from CPRD to Office of National Statistics Lower layer Super Output Area (ONS-LSOA). This contains patient-level deprivation data for patients in England (Office of National Statistics, 2015).

This study was approved by the Independent Scientific Advisory Committee of CPRD (protocol no. 18_288). Informed consent is waived because data are anonymized for research purposes.

### Study population

For our cohort study, we identified individuals with SMI diagnoses within CPRD using primary care Read codes for schizophrenia, bipolar disorder or other non-organic psychotic illnesses. ‘Other psychosis’ captures a range of psychotic disorders, including schizoaffective disorder, delusional disorders, acute or transient psychoses and unspecified psychoses (online Supplementary table 1, supplementary figure 1). “Other psychosis” codes are the most common codes used for recording psychosis in primary care (Hardoon et al., 2013), which may reflect hesitancy in giving stigmatizing diagnoses such as schizophrenia early in the illness (McGorry, Killackey, & Yung, 2008). We excluded psychosis related to substance misuse, as this group would be at risk of delirium due to substance withdrawal; a related, but different clinical phenomenon.

**Figure 1.**
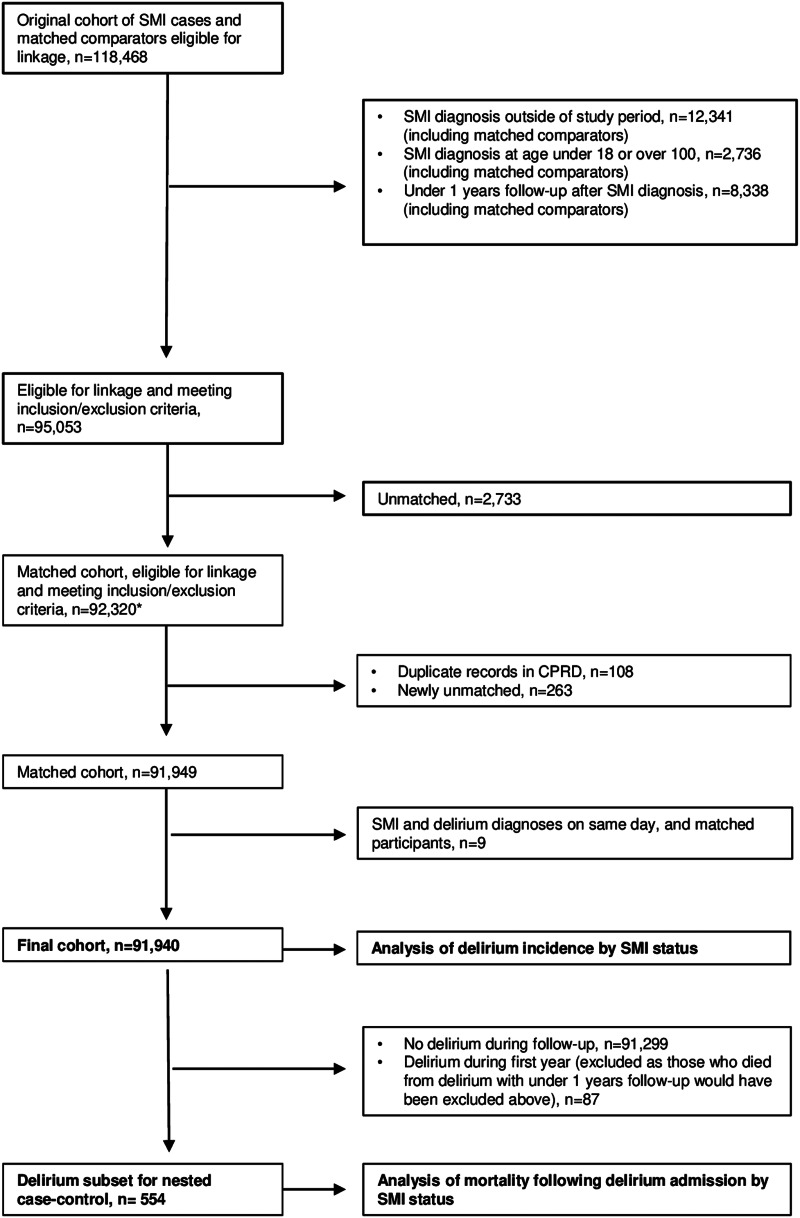
STROBE flow diagram of patients showing numbers and reasons for exclusion. *SMI, severe mental illness; CPRD, Clinical Practice Research Datalink.* *Population used for assessment of linkage bias (online Supplementary table 4).

We included people with a diagnosis of SMI between 1 April 2000 and 31 March 2016 to capture a minimum of one year's follow-up within HES data i.e. up to 31 March 2017. People with SMI diagnoses were matched to up to four comparators without SMI on five-year age band, sex, primary care practice and year of primary care practice registration. Matching was carried out by CPRD prior to receipt of the dataset. We excluded patients who were under age 18 or over age 100 at start of follow-up, those with less than one year of follow-up, and those without available matched comparators. We excluded those not eligible for HES data linkage; therefore our final cohort was from England only. In cases of a duplicate CPRD record, we included only the record with the earliest SMI diagnosis and excluded the duplicate, as this method has been previously validated (Sammon, Leahy, & Ramagopalan, 2020). We excluded people who received their SMI and delirium diagnoses on the same day, and their matched comparators, as these were likely due to miscoding, as delirium can be miscoded as an SMI (Otani et al., 2017) (Fig. 1, online Supplementary fig. 1). We stratified the cohort into younger (18–64 years) and older adults (⩾65 years) at cohort entry to assess whether SMI confers a differential risk for delirium diagnosis across the two age groups. We followed up patients from the date of their first SMI diagnosis (index date), and for comparators the same start date as their matched SMI patient, until the earliest of their first hospital delirium diagnosis, end of CPRD record, death or 31 March 2017.

For our nested case–control study, we included the subgroup of individuals who received a delirium diagnosis during cohort follow-up. As we excluded anyone with under one year's follow-up from index date from our cohort, patients who died following delirium within their first year would have been excluded. Therefore for the nested case–control study we included only delirium diagnoses coded at least one year after SMI diagnosis (or equivalent date) (Fig. 1).

### Outcomes

In our cohort study, the outcome was the first recorded delirium diagnosis, identified using ICD-10 codes in HES-APC for delirium or acute encephalopathy (Slooter et al., 2020) (online Supplementary table 3). We analyzed first delirium episode only to eliminate the risk conferred by prior delirium on subsequent delirium (Ormseth et al., 2023).

In our nested case–control study, cases were defined as those who had mortality recorded within 30 days of discharge date from a hospitalization involving delirium. Controls had a hospitalization involving delirium but no reported mortality within 30 days of discharge. Mortality data was taken from both HES (death within hospitalization) and CPRD (death within 30 days of hospital discharge), as this captures death at 98.8% accuracy for this time frame within CPRD (Gallagher, Dedman, Padmanabhan, Leufkens, & de Vries, 2019). Cases and controls were compared in terms of SMI status.

### Covariates

We collected covariate data at index date from CPRD and the ONS-LSOA databases, including age, sex, ethnicity, region, Index of Multiple Deprivation (IMD) quintile and physical comorbidities. We defined age at index date and age at hospital admission as continuous variables based on year of birth. Sex was reported as male or female as per primary care records. We categorized ethnicity as Asian, Black, White, Mixed or Other based on the UK 2011 Census Ethnic Group categories (Office for National Statistics, 2011). Region was based on primary care practice postcode, and IMD quintile by patient postcode. We collected data on physical comorbidities at index based on a count of 24 physical health conditions defined from code lists for the Charlson and Elixhauser comorbidity indices (Launders et al., 2022b) and categorized as 0,1 or ⩾1 at index date.

### Missing data

For missing ethnicity data, we used single imputation and re-classified missing ethnicity as ‘white’ in keeping with previously validated methods (Hippisley-Cox et al., 2008). Where patient postcode was missing, IMD quintile was defined by primary care practice postcode.

### Statistical analysis: delirium incidence

We investigated whether incidence of delirium diagnoses varied by SMI status using Cox proportional hazard regression. We tested the proportional hazards assumption using Schoenfeld residuals for unadjusted and adjusted models (online Supplementary figure 2). We performed Kaplan–Meier analysis to visually examine proportionality and obtain the predicted survival plots. We obtained unadjusted and adjusted hazard ratios (HRs and aHRs respectively) for delirium diagnosis incidence by SMI status, stratified according to age group at start of follow-up; younger (18–64 years) and older (⩾65 years) adults (online Supplementary figure 3). We adjusted for sex, ethnicity and deprivation level as potential confounders as they are associated with both the exposure (SMI) and outcome (delirium) (Arias et al., 2020; Choi et al., 2012; Khan et al., 2016; Ormseth et al., 2023). To account for possible changes in delirium coding over the follow-up period, including in people with SMI, we modelled calendar time as a time-varying covariate (in 2-year time bands) (Bauernfreund et al., 2022; Ibitoye et al., 2023a). We modelled age as a time-varying covariate in five-year age bands, as risk of delirium varies markedly with age (Pandharipande et al., 2006). We used a stratification term within the model due to possible similarities within primary care practices. We did not adjust for physical comorbidities or frequency of hospitalizations, as these are likely to represent an intermediary step on the causal pathway between SMI and delirium.

To examine the potential effect of misdiagnosis between SMI and delirium, we conducted sensitivity analyses with washout periods, excluding those who received a delirium diagnosis within three or six months of their initial SMI diagnosis. We chose these time points to reflect the duration of most cases of delirium (Wilson et al., 2020) as it is unlikely after six months that the SMI and delirium codes reflect the same presentation.

### Statistical analysis: mortality following delirium

As our nested case–control study included only patients diagnosed with delirium, this subset was no longer matched. We performed logistic regression and adjusted for age at hospital admission, physical comorbidities, sex, ethnicity, and deprivation level, as these factors could confound differences in mortality (Pessoa et al., 2020; Pocock, Ives, Pring, Verne, & Purdy, 2016). In this analysis, ethnicity was collapsed into two categories of white and minority ethnic groups due to small numbers in some ethnic categories. All statistical analyses were conducted using Stata-17.

### Subgroup analysis

As schizophrenia, bipolar disorder and ‘other psychoses’ are distinct conditions, we performed a-priori stratified subgroup analyses to assess whether risk of delirium diagnosis and 30-day mortality differed according to SMI subtype.

## Results

### Patient sample

91 940 individuals (20 566 people with SMI diagnoses and 71 374 matched comparators) met the inclusion criteria for our study (Fig. 1). Patients eligible for linkage had similar demographic characteristics and SMI diagnoses to those not eligible (online Supplementary table 4) (Launders et al., 2022a). 76 300 patients were in the younger age group (18–64 years) and 15 640 in the older age group (⩾65 years).

### Baseline characteristics

Across both age groups, patients with SMI diagnoses were more likely to be from the most deprived quintile and were more likely to die during follow-up than matched comparators (Table 1). In the younger age group, patients with SMI diagnoses were more likely to be from an ethnic minority group (15.2% v. 12.8%) and more likely to have at least one physical comorbidity (36.6% v. 30.9%) than matched comparators. In the older group, patients with SMI had shorter follow-up than matched comparators (3.45 v. 4.74 years) (Table 1).

**Table 1.** Baseline characteristics of cohort, stratified by age at start of follow-up (18–64 years v. ⩾65 years), by SMI status

	18-64 years (at start)		⩾65 years (at start)	
	No SMI	SMI	No SMI	SMI
N	59 208	17 092	12 166	3474
Follow-up time (years) (IQR)	4.89 (2.54–8.79)	4.56 (2.36–8.34)	4.74 (2.61–8.03)	3.45 (1.97–6.04)
Year of birth Median (IQR)	1970 (1960–1981)	1971 (1960–1981)	1930 (1924–1937)	1930 (1923–1936)
Age at index date* (IQR)	37.7 (27.8–48.1)	36.9 (27.5–47.4)	77.0 (70.8–83.4)	77.6 (71.1–84)
Female (%)	26 465 (44.7)	7677 (44.9)	8293 (68.2)	2382 (68.6)
Ethnicity (%)				
Asian	3648 (6.16)	985 (5.76)	276 (2.27)	92 (2.65)
Black	1951 (3.30)	937 (5.48)	192 (1.58)	61 (1.76)
Mixed	488 (0.82)	230 (1.35)	26 (0.21)	7 (0.20)
Other	1493 (2.52)	441 (2.58)	248 (2.04)	66 (1.90)
White**	51 628 (87.2)	14 499 (84.8)	11 424 (93.9)	3248 (93.5)
Region (%)				
East Midlands	1996 (3.37)	550 (3.22)	419 (3.44)	114 (3.28)
East of England	5133 (8.67)	1428 (8.35)	1122 (9.22)	312 (8.98)
London	12 131 (20.5)	3658 (21.4)	1718 (14.1)	503 (14.5)
North East	1150 (1.94)	318 (1.86)	321 (2.64)	89 (2.56)
North West	7954 (13.4)	2284 (13.4)	1511 (12.4)	436 (12.6)
South Central	8059 (13.6)	2341 (13.7)	1753 (14.4)	496 (14.3)
South EC	5689 (9.61)	1655 (9.68)	1172 (9.63)	341 (9.82)
South West	8011 (13.5)	2323 (13.6)	1976 (16.2)	569 (16.4)
West Midlands	6808 (11.5)	1907 (11.2)	1694 (13.9)	482 (13.9)
Y&TH	2277 (3.85)	628 (3.67)	480 (3.95)	132 (3.80)
IMD quintile (%) ***				
1 – least deprived	10 931 (18.5)	2384 (14.0)	2706 (22.2)	672 (19.3)
2	11 230 (19.0)	2620 (15.3)	2661 (21.9)	717 (20.6)
3	11 515 (19.5)	3175 (18.6)	2455 (20.2)	701 (20.2)
4	13 283 (22.4)	4112 (24.1)	2391 (19.7)	735 (21.2)
5 – most deprived	12 249 (20.7)	4801 (28.1)	1953 (16.1)	649 (18.7)
Physical comorbidities at index (%)				
None	48 882 (69.1)	10 830 (63.4)	2596 (21.3)	749 (21.6)
One	13 806 (23.3)	4414 (25.8)	3276 (26.9)	894 (25.7)
More than one	4520 (7.63)	1848 (10.8)	6294 (51.7)	1831 (52.7)
SMI diagnosis				
Schizophrenia	-	4033 (23.6)	-	520 (15.0)
Bipolar disorder	-	6462 (37.8)	-	992 (28.6)
Other Psychosis	−	6597 (38.6)	-	1962 (56.5)
Died (%)	1031 (1.74)	771 (4.51)	3503 (28.8)	1318 (37.9)

Continuous variables are displayed as median (IQR). Categorical variables are displayed as n(%),to 3 significant figures

*Age at index date is age on date of SMI diagnosis, and for matched comparators is age on the date their matched SMI case was diagnosed. *SMI, severe mental illness; IQR, interquartile range; IMD, index of multiple deprivation.*

**Missing ethnicity data coded as white [SMI: 7885/20 566 (38.3%), No SMI: 30 923/71 374 (43.3%)].

***Missing patient IMD data coded as primary care practice IMD [SMI: 44/20 566 (0.21%), No SMI: 102/71 374 (0.14%)].

### Incidence of delirium diagnosis by SMI status

641 (0.7%) individual patients had a recorded hospital delirium diagnosis during follow-up. The overall incidence of delirium diagnoses in people with SMI was 2.44 cases per 1000 person-years (95% CI 2.16–2.74) compared to 0.85 per 1000 person-years (95% CI 0.76–0.94) in matched comparators. In both people with and without SMI diagnoses, the incidence of delirium diagnoses was higher in the older age group than in the younger age group (Table 2). In the younger group, people with SMI diagnoses were over seven times more likely to have a recorded delirium diagnosis than matched comparators [incidence 1.00 (95% CI 0.82–1.21) per 1000 person-years v. 0.15 (95% CI 0.11–0.19) per 1000 person-years; aHR:7.65, (95% CI 5.45–10.7, *p* < 0.001)] whereas in the older age group people with SMI were more than three times as likely to have a recorded delirium diagnosis [incidence 11.6 (95% CI 10.0–13.44) per 1000 person-years v. 4.48 (95% CI 4.01–5.01) per 1000 person-years; aHR:3.35, (95% CI 2.77–4.05, *p* < 0.001)] (Table 2, online Supplementary figure 3).

**Table 2.** Incidence and hazard ratios for delirium diagnosis by SMI status and subtype

Age group		Person-years	Recorded delirium diagnoses (first)	Incidence rate (per 1000 person-years) (95% CI)	Association of SMI Unadjusted HR (95% CI)	*p*-value	Association of SMI Adjusted HR* (95% CI)	*p*-value
Whole cohort	No SMI	426 676.44	363	0.85 (0.76–0.94)	1 (ref)		1 (ref)	
	SMI	113 925.92	278	2.44 (2.16–2.74)	2.92 (2.50–3.42)	<0.001	4.00 (3.43–4.67)	<0.001
18-64 years	No SMI	357 309.59	52	0.15 (0.11–0.19)	1 (ref)		1 (ref)	
	SMI	98 426.84	98	1.00 (0.82–1.21)	6.98 (4.99–9.77)	<0.001	7.65 (5.45–10.7)	<0.001
	Scz	24 602.80	18	0.73 (0.46–1.16)	4.92 (2.88–8.41)	<0.001	5.77 (3.38–9.85)	<0.001
	BPAD	37 601.52	37	0.98 (0.71–1.36)	6.90 (4.53–10.5)	<0.001	7.38 (4.76–11.5)	<0.001
	Other	36 222.52	43	1.19 (0.89–1.60)	8.67 (5.72–12.8)	<0.001	9.18 (6.17–13.7)	<0.001
⩾65 years	No SMI	69 366.85	311	4.48 (4.01–5.01)	1 (ref)		1 (ref)	
	SMI	15 499.08	180	11.6 (10.0–13.44)	2.85 (2.37–3.44)	<0.001	3.35 (2.77–4.05)	<0.001
	Scz	2474.80	21	8.49 (5.53–13.01)	2.02 (1.30–3.14)	0.002	2.96 (1.90–4.60)	<0.001
	BPAD	5342.85	56	10.48 (8.07–13.62)	2.42 (1.82–3.22)	<0.001	3.93 (2.89–5.35)	<0.001
	Other	7681.43	103	13.41 (11.05–16.27)	3.52 (2.81–4.42)	<0.001	3.16 (2.48–4.03)	<0.001

Incidence rates, hazard ratios and confidence intervals reported to 2 decimal places. *Adjusted HRs are adjusted for age, calendar year, sex, ethnicity, deprivation level, with age and calendar year modelled as time-varying covariates. *SMI, severe mental illness; Scz*, *schizophrenia; BPAD, Bipolar Affective disorder; Other, Other Psychosis; HR, hazard ratio; CI, confidence interval; ref, reference category for hazard ratios.*

In the younger group, female patients had lower rate of delirium diagnoses (aHR 0.65, 95% CI 0.45-0.94, *p* = 0.022), and patients of mixed ethnicity had higher rate of delirium diagnoses (aHR 4.91, 95% CI 1.51–16.0, *p* = 0.008). In the older group, gender and ethnicity did not significantly affect risk of delirium diagnosis. In both age groups, patients residing in the most deprived areas (IMD quintiles) had higher rate of delirium diagnoses (younger group: most deprived quintile aHR 2.98 (95% CI 1.45–6.12, *p* = 0.003), older group: most deprived quintile aHR 1.58 (95% CI 1.08–2.30, *p* = 0.017) (online Supplementary table 7).

### Incidence of delirium diagnosis by SMI subtype

When stratified by SMI subtype, in the younger age group, risk of delirium diagnosis was highest in the ‘other psychosis’ group (aHR:9.18, 95% CI 6.17–13.7, *p* < 0.001), followed by bipolar disorder (aHR:7.38, 95% CI 4.76–11.5, *p* < 0.001), and then schizophrenia (aHR:5.77, 95% CI 3.38–9.85, *p* < 0.001), compared to people without SMI (Table 2, online Supplementary figure 3). In the older group, risk of hospital delirium diagnosis was highest in the ‘other psychosis’ group in the unadjusted model but highest for bipolar disorder in the adjusted model (aHR:3.93, 95% CI 2.89–5.35, *p* < 0.001), followed by ‘other psychosis’ (aHR:3.16, 95% CI 2.48–4.03, *p* < 0.001), then schizophrenia (aHR:2.96, 95% CI 1.90–4.60, *p* < 0.001). In all analyses, confidence intervals overlapped between SMI subtypes (Table 2).

### Sensitivity analysis: applying three and six month washout periods

When excluding patients who received SMI and delirium diagnoses within three months and six months of each other to account for possible misdiagnosis of delirium as SMI, effect sizes reduced slightly, but the association between SMI and delirium diagnoses remained significant (online Supplementary table 5).

### 30-day mortality following delirium

Of the 554 (0.6%) patients who received a hospital delirium diagnosis after their first year of follow-up, 85 (15.3%) died within 30 days of discharge (online Supplementary table 6). Patients who died within 30 days of a hospitalization involving delirium diagnosis were older, more deprived and more physically co-morbid than the full cohort and delirium subgroup (online Supplementary table 6).

### Odds of 30-day mortality by SMI status and subtype

In our adjusted logistic regression, there was no significant difference in 30-day mortality following discharge from hospitalization involving delirium diagnosis between people with SMI diagnoses and comparators (aOR:0.66, 95% CI 0.38–1.14, *p* = 0.135) (Table 3). When analyzed by subtype, people with schizophrenia and bipolar disorder had no significant difference in odds of mortality compared to those without SMI. Patients with ‘other psychosis’ had reduced odds of death (aOR:0.31, 95% CI 0.13–0.76, *p* = 0.011) (Table 3).

**Table 3.** Odds of mortality within 30 days of hospitalization involving a delirium diagnosis by SMI status and subtype

	*N*	*n* died within 30 days of delirium (%)	Unadjusted OR (95% CI)	*p*-value	Adjusted* OR (95% CI)	*p*-value
No SMI	344	61 (17.7)	1 (ref)		1 (ref)	
SMI	210	24 (11.4)	0.59 (0.36–0.99)	0.047	0.66 (0.38–1.14)	0.135
Schizophrenia	33	4 (12.1)	0.64 (0.22–1.89)	0.418	0.81 (0.26–2.51)	0.715
Bipolar Disorder	79	14 (17.7)	1.00 (0.53–1.90)	0.998	1.18 (0.59–2.34)	0.640
Other Psychosis	98	6 (6.12)	0.30 (0.13–0.72)	0.007	0.31 (0.13–0.76)	0.011

*Adjusted for sex, ethnicity, deprivation level, age at hospital admission and physical comorbidities. Total N for each analysis is displayed. ORs are reported to 2 decimal places. *SMI, severe mental illness; OR, odds ratio; CI, confidence interval; ref, reference category for hazard ratios.*

## Discussion

### Summary of findings

Our study presents analyses of rates of delirium diagnoses in people with SMI diagnoses compared to people without. We found that people with pre-existing SMI diagnoses, including schizophrenia, bipolar disorder and other psychotic disorders had a higher incidence of recorded hospital delirium diagnoses than matched comparators. Although there was a higher rate of delirium diagnoses in the older age group (⩾65 years at cohort entry), the relative delirium risk conferred by having SMI was larger in the younger group (18–64 years at cohort entry). Compared to matched comparators without SMI, younger adults with SMI had over seven times the risk of delirium diagnosis and older adults with SMI had over three times the risk of delirium diagnosis.

In subgroup analyses of delirium incidence, we found that in the younger group, patients with ‘other psychosis’ had the highest rate of delirium diagnoses, while in the older group those with bipolar disorder had the highest rate. Given its broader definition, ‘other psychosis’ could include more misclassified delirium or organic psychoses predisposing to delirium. A high rate of delirium in people with bipolar disorder compared to other psychiatric subgroups has been noted in a previous study (Ritchie, Steiner, & Abrahamowicz, 1996). Notably lithium, a medication used for bipolar disorder, can cause neuro-toxicity and a delirium-like syndrome, however in that study, patients with bipolar disorder had higher risk of delirium regardless of lithium prescription (Ritchie et al., 1996). Patients with bipolar disorder are more likely to have planned physical health hospital admissions than patients with schizophrenia (Launders et al., 2022a), thus may be more exposed to precipitants for delirium such as planned surgeries. Patients with bipolar disorder may have an inherent vulnerability to delirium; relapses may impact food and fluid intake and cause electrolyte disturbances (Hochman, Weizman, Valevski, Fischel, & Krivoy, 2014) which may precipitate delirium (Ormseth et al., 2023).

Perhaps surprisingly, we found no difference between those with SMI and those without in 30-day mortality following discharge from hospitalization involving delirium diagnosis. However, given we found a higher incidence of delirium diagnosis in people with SMI, similar odds of mortality from delirium to people without SMI would still amount to substantial delirium mortality in this group. Reduced odds of mortality in the ‘other psychosis’ subgroup is more difficult to interpret, and may reflect less fatal delirium caused by smaller physiological insults as seen in more frail groups in other studies (Dani et al., 2017; Sahle et al., 2022).

### Comparison to other studies

Previous studies investigating the relative risk of delirium in populations with SMI are sparse. The overall incidence of delirium diagnoses in people with SMI in this cohort was 0.24 cases per 100 person-years. This is lower than the annual incidence in 2017 in a cohort of people with SMI as we reported previously; 1.05 per 100 person-years (Bauernfreund et al., 2022), likely due to the incidence for this cohort being calculated over the whole period from 2000–2017, over which incidence markedly increased (Bauernfreund et al., 2022). It is also lower than absolute incidence of delirium reported previously in a Danish psychiatric cohort, 0.84 per 100 person-years (Lundberg et al., 2014b); however delirium assessment is known to vary widely across different countries (Nydahl et al., 2024).

Three recent studies using EHRs report similar findings to this study. A case–control study using CPRD-HES data to build a delirium prediction model for the community found ‘serious mental illness’ to confer almost 7-fold higher odds of delirium (Bowman et al., 2020); similar to our study. A USA study examining comorbidities for schizophrenia patients found a higher risk of delirium than for comparators (Lu et al., 2022). A retrospective study of risk factors for delirium following elective hip arthroplasty using a Chinese database found a history of psychosis to increase risk (Yang, Wang, Huang, Xu, and Zhang, 2020a). However, unlike our study, these studies were not focused on testing an association between SMI and delirium diagnoses.

Findings from observational studies in hospital cohorts are inconsistent, numbers of patients with SMI are small, and the term ‘psychiatric illness’ is usually broadly defined. A meta-analysis pooling 17 studies examining risk factors for delirium after colorectal cancer surgery found a history of psychiatric illness increased odds of post-operative delirium 6-fold, although most had a background of depression (Yang et al., 2020b). Another recent systematic review found broadly defined psychiatric disorders to be a predisposing factor for delirium (Ormseth et al., 2023), and a systematic review for risk factors for post-operative delirium after non-cardiac surgery found psychopathological symptoms to correlate with likelihood of delirium (Dasgupta & Dumbrell, 2006).

Our finding that a greater difference in risk of delirium diagnosis was seen between people with SMI and without at younger ages is similar to that seen in studies of multimorbidity; although an increased risk is seen in SMI at all ages, the difference from comparators is greatest at younger ages (Launders et al., 2022b). People with SMI age prematurely and experience frailty at younger ages (Lin et al., 2021; Pearson et al., 2022) thus a greater difference is seen in the younger age group when delirium is rare in the general population.

It is interesting to note that in studies of coding trends within American large datasets over a similar timeframe (2011–2018), acute encephalopathy is coded more frequently than delirium (Franks et al., 2022), whereas in this study we found only 7% of total outcome codes for acute encephalopathy (online Supplementary table 3). This difference between UK and US may be due to higher reimbursement from US insurance companies for coding of ‘acute encephalopathy’ than ‘delirium’, leading to US hospital financial incentivisation to code ‘acute encephalopathy’ (Epps & Tong, 2018). International differences in coding of the same syndrome in clinical data and current literature presents an obstacle to progressing clinical care and research, and more unified terminology defined by consensus is needed (Slooter et al., 2020).

Our 30-day mortality rates following hospitalization involving delirium for people without SMI and the bipolar disorder group were similar to 30-day mortality rates reported in other recent studies, around 17% (Anand, Cheng, Ibitoye, Maclullich, & Vardy, 2022; Arneson et al., 2023). We found lower mortality rates in people with ‘other psychosis’. There is scarce other literature reporting delirium mortality in patients with SMI. Interestingly, a study examining predictors of delirium mortality in older people receiving mental health-care showed no association between mortality and psychotic, agitated or depressive symptoms (Ward, Perera, & Stewart, 2015). Previous work has shown that delirium's impact on mortality is actually lower at higher levels of frailty (Dani et al., 2017), and a recent study showed that although delirium is more common in frail patients, it does not modify the association between frailty and in-hospital mortality (Sahle et al., 2022). This may be because the physiological insult needed to cause delirium in more frail individuals is smaller (Inouye, 1999), so fatality is lower.

The ‘other psychosis subgroup’ may be more frail than the other two subgroups. Age onset of ‘other psychosis’ in males follows that seen in schizophrenia, but in females is most common over the age of 75 (Hardoon et al., 2013). There is an established association between very-late onset psychosis and dementia in routine data, and it may be that a proportion of patients with ‘other psychosis’ codes are actually experiencing prodromal changes of dementia (Stafford et al., 2023). In addition, the ‘other psychosis’ group had more multimorbidity than the other subgroups (online Supplementary table 2). Thus the ‘other psychosis’ group may be more physically and cognitively frail, and this may explain why delirium-related mortality is lower. Alternatively, this group may have more misclassified or mis-coded delirium rather than a definite SMI, causing falsely low mortality rates. Lundberg et al., demonstrated that delirium increases mortality rate for psychiatric patients (Lundberg, Gustafsson, Meagher, & Munk-Jørgensen, 2014a); therefore although delirium mortality rates may be lower for people with ‘other psychosis’ than the general population, delirium in this group may still be associated with a poorer prognosis.

### Strengths & limitations

To our knowledge, this is a novel study designed to test whether people with SMI diagnoses have higher rates of delirium diagnoses than people without SMI, using a nationally representative, longitudinal cohort. This comparison to rates of delirium diagnoses in the general population builds on our earlier analyses (Bauernfreund et al., 2022) to provide a robust insight into the question of relative risk of delirium diagnoses in people with SMI.

Our delirium estimates are conservative, as delirium is under-detected in clinical coding (Ibitoye et al., 2023b) so picking up false positive cases is unlikely. As delirium is often misdiagnosed, miscoding initially as a psychotic disorder would be more likely (Otani et al., 2017; Swigart, Kishi, Thurber, Kathol, & Meller, 2008). We examined this by performing a sensitivity analysis using three and six month washout periods. This resulted in small reductions in our effect sizes, however the association between SMI and delirium diagnoses remained highly significant, suggesting misdiagnosis was unlikely to fully explain the association. We adjusted for demographic variables which may confound the association between SMI and delirium, including sex, ethnicity and deprivation, and modelled age and calendar year as time-varying covariates. People with SMI have a reduced life-expectancy (Public Health England, 2018), as was found in our study in both younger and older age groups (Table 1), yet they still experienced a higher incidence of delirium diagnosis. Thus effect size may be underestimated due to survivorship bias.

Our study has several limitations. We used linked data from CPRD only, restricting the study to England rather than UK-wide. There have been national efforts to improve delirium awareness in Scotland (Health Improvement Scotland, 2017) and excluding this region may have under-estimated delirium incidence. In addition, we used an early CPRD Aurum dataset (Wolf et al., 2019) with comparatively low levels of linkage compared to later datasets (CPRD, 2024a). We ascertained delirium diagnoses from hospital data only, so may have missed more mild delirium diagnosed in the community. We did not differentiate between delirium on general wards and delirium in intensive care units (ICUs), where it is highly prevalent (Ely et al., 2001). There is little literature on SMI and intensive-care use, however disparities in ICU admission for people with schizophrenia were reported during the COVID-19 pandemic (Fond et al., 2021). We do not know whether disparities in ICU admission for people with SMI occurred prior to COVID-19 i.e. during our study period, and affected rates of delirium diagnoses for this group.

There are factors which could lead to differential hospital delirium recording in people with SMI and affect the validity of our findings. People with SMI are more likely to attend hospital (Launders et al., 2022a), so would be more likely to experience hospital delirium. Once in general hospital, people with SMI are seen by liaison psychiatry services, who are more likely to detect delirium than general medical or surgical teams, leading to outcome bias (Swigart et al., 2008). We did not adjust for overall hospitalization rate as we wanted to address the question of whether people with SMI experience more delirium, regardless of the pathway to this. Similarly, we did not adjust for physical comorbidities, antipsychotic medications or frailty in our primary analysis, as these factors are likely to lie on the causal pathway between SMI and delirium. Our aim was to assess relative rates of delirium diagnoses in SMI compared to the general population, and exploring the role of these factors introduces complexity which should be analyzed in depth in separate studies.

We did not collect data on dementia diagnosis. Evidence suggests psychotic disorders and bipolar disorder increase the risk of dementia by 2–3 times (Miniawi, Orgeta, & Stafford, 2022; Velosa et al., 2020). Dementia is a long-established key pre-disposing factor for delirium (Inouye, 1999), and recent evidence demonstrates that this relationship is bi-directional, and delirium is a risk factor for dementia (Richardson et al., 2020; Tsui et al., 2022). Thus the interplay between cognitive impairment in SMI, dementia and delirium is complex, and deserves careful analysis in separate studies.

The results of our mortality analysis are more difficult to interpret. This analysis was performed in non-matched participants. We aimed to account for this by adjusting for age at admission, sex, ethnicity, deprivation and physical comorbidities. In our adjusted model, our finding of SMI conferring a reduced odds of mortality was no longer significant, and it may be that adjustment reduced the power in our model. Alternatively, adjusting for these confounders may have revealed a true lack of association between SMI and delirium-related mortality. We note wide confidence intervals in our effect estimates for mortality, and our lack of significant finding may be due to the small sample size for this analysis.

### Clinical and research implications

Given SMI diagnosis predisposes to delirium diagnosis, further prospective studies are needed to explore underlying mechanisms for this association. Our previous work demonstrated that in people with SMI, delirium is associated with older age, more physical comorbidity and more antipsychotic medication (Bauernfreund et al., 2022). Future studies could investigate whether these clinical variables could be used to develop risk prediction models for delirium in this population, and whether a comprehensive multi-factorial assessment could prevent delirium in this group, as has been demonstrated for general older populations (Mueller, Street, Carnahan, & Lee, 2023; Shields, Henderson, & Caslake, 2017). This study highlights that further research is needed into factors driving delirium in younger patients; among which people with SMI experience a serious inequality. Furthermore, we must improve our understanding of the clinical presentation of delirium in this group who are at risk of diagnostic overshadowing, to enable timely recognition and treatment of underlying physiological precipitants. Further studies are needed to explore rates of other complications of delirium in this group, including long-term cognitive effects and healthcare costs.

Our findings have several important clinical implications. If prospective studies replicate the association between SMI and delirium, regular screening and prevention strategies may be needed for people with SMI in hospital, as for other groups with delirium vulnerability (NICE, 2010). We need staff training, in both general and psychiatric hospitals, to improve detection of delirium in younger people with SMI who may be much more prone than in the general population. The National Institute for Clinical Excellence (NICE) acknowledges that people with pre-existing SMI are at risk of being treated less favorably because changes in their mental state are often attributed to their existing condition rather than to delirium (NICE, 2023). If people with SMI have higher risk of delirium, it is all the more important that we address this inequality by improving our understanding, recognition and treatment of delirium in this group.

## Supporting information

Bauernfreund et al. supplementary material Bauernfreund et al. supplementary material

## Supplementary material

The supplementary material for this article can be found at https://doi.org/10.1017/S0033291724002484.
