# Supplementary material for: Delirium risk and mortality in people with pre-existing severe mental illness: a retrospective cohort study using linked datasets in England
Source: Psychol Med. 2024 Oct 31;54(14):3974–84. doi: 10.1017/S0033291724002484 (PMC11578903; doi:10.1017/S0033291724002484)
Supplement: Bauernfreund et al. supplementary material [file S0033291724002484sup001.docx]

Supplementary material

Supplementary Table 1 – SMI Read code lists

| **Read code** | **Term** | **Group** |
| --- | --- | --- |
| E110 | Manic disorder, single episode | bipolar |
| E110-1 | Hypomanic psychoses | bipolar |
| E110-99 | Mania/hypomania | bipolar |
| E1100 | Single manic episode, unspecified | bipolar |
| E1101 | Single manic episode, mild | bipolar |
| E1102 | Single manic episode, moderate | bipolar |
| E1103 | Single manic episode, severe without mention of psychosis | bipolar |
| E1104 | Single manic episode, severe, with psychosis | bipolar |
| E1105 | Single manic episode in partial or unspecified remission | bipolar |
| E1106 | Single manic episode in full remission | bipolar |
| E110z | Manic disorder, single episode NOS | bipolar |
| E111 | Recurrent manic episodes | bipolar |
| E1110 | Recurrent manic episodes, unspecified | bipolar |
| E1111 | Recurrent manic episodes, mild | bipolar |
| E1112 | Recurrent manic episodes, moderate | bipolar |
| E1113 | Recurrent manic episodes, severe without mention psychosis | bipolar |
| E1114 | Recurrent manic episodes, severe, with psychosis | bipolar |
| E1115 | Recurrent manic episodes, partial or unspecified remission | bipolar |
| E1116 | Recurrent manic episodes, in full remission | bipolar |
| E111z | Recurrent manic episode NOS | bipolar |
| E114 | Bipolar affective disorder, currently manic | bipolar |
| E114-1 | Manic-depressive - now manic | bipolar |
| E1140 | Bipolar affective disorder, currently manic, unspecified | bipolar |
| E1141 | Bipolar affective disorder, currently manic, mild | bipolar |
| E1142 | Bipolar affective disorder, currently manic, moderate | bipolar |
| E1143 | Bipolar affect disord, currently manic, severe, no psychosis | bipolar |
| E1144 | Bipolar affect disord, currently manic,severe with psychosis | bipolar |
| E1145 | Bipolar affect disord,currently manic, part/unspec remission | bipolar |
| E1146 | Bipolar affective disorder, currently manic, full remission | bipolar |
| E114z | Bipolar affective disorder, currently manic, NOS | bipolar |
| E115 | Bipolar affective disorder, currently depressed | bipolar |
| E115-1 | Manic-depressive - now depressed | bipolar |
| E1150 | Bipolar affective disorder, currently depressed, unspecified | bipolar |
| E1151 | Bipolar affective disorder, currently depressed, mild | bipolar |
| E1152 | Bipolar affective disorder, currently depressed, moderate | bipolar |
| E1153 | Bipolar affect disord, now depressed, severe, no psychosis | bipolar |
| E1154 | Bipolar affect disord, now depressed, severe with psychosis | bipolar |
| E1155 | Bipolar affect disord, now depressed, part/unspec remission | bipolar |
| E1156 | Bipolar affective disorder, now depressed, in full remission | bipolar |
| E115z | Bipolar affective disorder, currently depressed, NOS | bipolar |
| E116 | Mixed bipolar affective disorder | bipolar |
| E1160 | Mixed bipolar affective disorder, unspecified | bipolar |
| E1161 | Mixed bipolar affective disorder, mild | bipolar |
| E1162 | Mixed bipolar affective disorder, moderate | bipolar |
| E1163 | Mixed bipolar affective disorder, severe, without psychosis | bipolar |
| E1164 | Mixed bipolar affective disorder, severe, with psychosis | bipolar |
| E1165 | Mixed bipolar affective disorder, partial/unspec remission | bipolar |
| E1166 | Mixed bipolar affective disorder, in full remission | bipolar |
| E116z | Mixed bipolar affective disorder, NOS | bipolar |
| E117 | Unspecified bipolar affective disorder | bipolar |
| E1170 | Unspecified bipolar affective disorder, unspecified | bipolar |
| E1171 | Unspecified bipolar affective disorder, mild | bipolar |
| E1172 | Unspecified bipolar affective disorder, moderate | bipolar |
| E1173 | Unspecified bipolar affective disorder, severe, no psychosis | bipolar |
| E1174 | Unspecified bipolar affective disorder,severe with psychosis | bipolar |
| E1175 | Unspecified bipolar affect disord, partial/unspec remission | bipolar |
| E1176 | Unspecified bipolar affective disorder, in full remission | bipolar |
| E117z | Unspecified bipolar affective disorder, NOS | bipolar |
| E11y | Other and unspecified manic-depressive psychoses | bipolar |
| E11y0 | Unspecified manic-depressive psychoses | bipolar |
| E11y1 | Atypical manic disorder | bipolar |
| E11y3 | Other mixed manic-depressive psychoses | bipolar |
| E11yz | Other and unspecified manic-depressive psychoses NOS | bipolar |
| E11z | Other and unspecified affective psychoses | bipolar |
| E11z0 | Unspecified affective psychoses NOS | bipolar |
| E11zz | Other affective psychosis NOS | bipolar |
| Eu30 | [X]Manic episode | bipolar |
| Eu30-1 | [X]Bipolar disorder, single manic episode | bipolar |
| Eu300 | [X]Hypomania | bipolar |
| Eu301 | [X]Mania without psychotic symptoms | bipolar |
| Eu302 | [X]Mania with psychotic symptoms | bipolar |
| Eu302-1 | [X]Mania with mood-congruent psychotic symptoms | bipolar |
| Eu302-2 | [X]Mania with mood-incongruent psychotic symptoms | bipolar |
| Eu302-3 | [X]Manic stupor | bipolar |
| Eu30y | [X]Other manic episodes | bipolar |
| Eu30z | [X]Manic episode, unspecified | bipolar |
| Eu30z-1 | [X]Mania NOS | bipolar |
| Eu31 | [X]Bipolar affective disorder | bipolar |
| Eu31-1 | [X]Manic-depressive illness | bipolar |
| Eu31-2 | [X]Manic-depressive psychosis | bipolar |
| Eu31-3 | [X]Manic-depressive reaction | bipolar |
| Eu310 | [X]Bipolar affective disorder, current episode hypomanic | bipolar |
| Eu311 | [X]Bipolar affect disorder cur epi manic wout psychotic symp | bipolar |
| Eu312 | [X]Bipolar affect disorder cur epi manic with psychotic symp | bipolar |
| Eu313 | [X]Bipolar affect disorder cur epi mild or moderate depressn | bipolar |
| Eu314 | [X]Bipol aff disord, curr epis sev depress, no psychot symp | bipolar |
| Eu315 | [X]Bipolar affect dis cur epi severe depres with psyc symp | bipolar |
| Eu316 | [X]Bipolar affective disorder, current episode mixed | bipolar |
| Eu317 | [X]Bipolar affective disorder, currently in remission | bipolar |
| Eu318 | [X]Bipolar affective disorder type I | bipolar |
| Eu31y | [X]Other bipolar affective disorders | bipolar |
| Eu31y-1 | [X]Bipolar II disorder | bipolar |
| Eu31y-2 | [X]Recurrent manic episodes | bipolar |
| Eu31z | [X]Bipolar affective disorder, unspecified | bipolar |
| Eu333-2 | [X]Manic-depress psychosis,depressed type+psychotic symptoms | bipolar |
| E11 | Affective psychoses | bipolar |
| E11-1 | Bipolar psychoses | bipolar |
| E11-99 | Manic-depressive psychoses | bipolar |
| Eu3z-1 | [X]Affective psychosis NOS | bipolar |
| 146D | H/O: manic depressive disorder | bipolar |
| 1S42 | Manic mood | bipolar |
| ZV111-1 | [V]Personal history of manic-depressive psychosis | bipolar |
| ZV111-2 | [V]Personal history of manic-depressive psychosis | bipolar |
| Eu332-3 | [X]Manic-depress psychosis,depressd,no psychotic symptoms | bipolar |
| E104-1 | Oneirophrenia | other psychosis |
| E105 | Latent schizophrenia | other psychosis |
| E1050 | Unspecified latent schizophrenia | other psychosis |
| E1051 | Subchronic latent schizophrenia | other psychosis |
| E1052 | Chronic latent schizophrenia | other psychosis |
| E1053 | Acute exacerbation of subchronic latent schizophrenia | other psychosis |
| E1054 | Acute exacerbation of chronic latent schizophrenia | other psychosis |
| E1055 | Latent schizophrenia in remission | other psychosis |
| E105z | Latent schizophrenia NOS | other psychosis |
| E107-1 | Cyclic schizophrenia | other psychosis |
| E107-99 | Acute schizo affective psychosis | other psychosis |
| E12 | Paranoid states | other psychosis |
| E12-99 | Paranoia | other psychosis |
| E120 | Simple paranoid state | other psychosis |
| E121 | Chronic paranoid psychosis | other psychosis |
| E121-1 | Sander's disease | other psychosis |
| E122 | Paraphrenia | other psychosis |
| E123 | Shared paranoid disorder | other psychosis |
| E123-1 | Folie a deux | other psychosis |
| E12y | Other paranoid states | other psychosis |
| E12y0 | Paranoia querulans | other psychosis |
| E12yz | Other paranoid states NOS | other psychosis |
| E12z | Paranoid psychosis NOS | other psychosis |
| E13 | Other nonorganic psychoses | other psychosis |
| E13-1 | Reactive psychoses | other psychosis |
| E131 | Acute hysterical psychosis | other psychosis |
| E133 | Acute paranoid reaction | other psychosis |
| E133-1 | Bouffee delirante | other psychosis |
| E134 | Psychogenic paranoid psychosis | other psychosis |
| E13y | Other reactive psychoses | other psychosis |
| E13y0 | Psychogenic stupor | other psychosis |
| E13y1 | Brief reactive psychosis | other psychosis |
| E13yz | Other reactive psychoses NOS | other psychosis |
| E13z | Nonorganic psychosis NOS | other psychosis |
| E13z-1 | Psychotic episode NOS | other psychosis |
| Eu2 | [X]Schizophrenia, schizotypal and delusional disorders | other psychosis |
| Eu21 | [X]Schizotypal disorder | other psychosis |
| Eu21-1 | [X]Latent schizophrenic reaction | other psychosis |
| Eu21-2 | [X]Borderline schizophrenia | other psychosis |
| Eu21-3 | [X]Latent schizophrenia | other psychosis |
| Eu21-4 | [X]Prepsychotic schizophrenia | other psychosis |
| Eu21-5 | [X]Prodromal schizophrenia | other psychosis |
| Eu21-6 | [X]Pseudoneurotic schizophrenia | other psychosis |
| Eu21-7 | [X]Pseudopsychopathic schizophrenia | other psychosis |
| Eu21-8 | [X]Schizotypal personality disorder | other psychosis |
| Eu22 | [X]Persistent delusional disorders | other psychosis |
| Eu220 | [X]Delusional disorder | other psychosis |
| Eu220-1 | [X]Paranoid psychosis | other psychosis |
| Eu220-2 | [X]Paranoid state | other psychosis |
| Eu220-3 | [X]Paraphrenia - late | other psychosis |
| Eu220-4 | [X]Sensitiver Beziehungswahn | other psychosis |
| Eu220-5 | [X]Paranoia | other psychosis |
| Eu221 | [X]Delusional misidentification syndrome | other psychosis |
| Eu221-1 | [X]Capgras syndrome | other psychosis |
| Eu22y | [X]Other persistent delusional disorders | other psychosis |
| Eu22y-1 | [X]Delusional dysmorphophobia | other psychosis |
| Eu22y-2 | [X]Involutional paranoid state | other psychosis |
| Eu22y-3 | [X]Paranoia querulans | other psychosis |
| Eu22z | [X]Persistent delusional disorder, unspecified | other psychosis |
| Eu23 | [X]Acute and transient psychotic disorders | other psychosis |
| Eu230 | [X]Acute polymorphic psychot disord without symp of schizoph | other psychosis |
| Eu230-1 | [X]Bouffee delirante | other psychosis |
| Eu230-2 | [X]Cycloid psychosis | other psychosis |
| Eu231 | [X]Acute polymorphic psychot disord with symp of schizophren | other psychosis |
| Eu231-1 | [X]Bouffee delirante with symptoms of schizophrenia | other psychosis |
| Eu231-2 | [X]Cycloid psychosis with symptoms of schizophrenia | other psychosis |
| Eu232 | [X]Acute schizophrenia-like psychotic disorder | other psychosis |
| Eu232-1 | [X]Brief schizophreniform disorder | other psychosis |
| Eu232-2 | [X]Brief schizophrenifrm psych | other psychosis |
| Eu232-3 | [X]Oneirophrenia | other psychosis |
| Eu233 | [X]Other acute predominantly delusional psychotic disorders | other psychosis |
| Eu233-2 | [X]Psychogenic paranoid psychosis | other psychosis |
| Eu23y | [X]Other acute and transient psychotic disorders | other psychosis |
| Eu23z | [X]Acute and transient psychotic disorder, unspecified | other psychosis |
| Eu23z-1 | [X]Brief reactive psychosis NOS | other psychosis |
| Eu23z-2 | [X]Reactive psychosis | other psychosis |
| Eu24 | [X]Induced delusional disorder | other psychosis |
| Eu24-1 | [X]Folie a deux | other psychosis |
| Eu24-2 | [X]Induced paranoid disorder | other psychosis |
| Eu24-3 | [X]Induced psychotic disorder | other psychosis |
| Eu25 | [X]Schizoaffective disorders | other psychosis |
| Eu250 | [X]Schizoaffective disorder, manic type | other psychosis |
| Eu250-1 | [X]Schizoaffective psychosis, manic type | other psychosis |
| Eu250-2 | [X]Schizophreniform psychosis, manic type | other psychosis |
| Eu251 | [X]Schizoaffective disorder, depressive type | other psychosis |
| Eu251-1 | [X]Schizoaffective psychosis, depressive type | other psychosis |
| Eu251-2 | [X]Schizophreniform psychosis, depressive type | other psychosis |
| Eu252 | [X]Schizoaffective disorder, mixed type | other psychosis |
| Eu252-1 | [X]Cyclic schizophrenia | other psychosis |
| Eu252-2 | [X]Mixed schizophrenic and affective psychosis | other psychosis |
| Eu25y | [X]Other schizoaffective disorders | other psychosis |
| Eu25z | [X]Schizoaffective disorder, unspecified | other psychosis |
| Eu25z-1 | [X]Schizoaffective psychosis NOS | other psychosis |
| Eu2y | [X]Other nonorganic psychotic disorders | other psychosis |
| Eu2y-1 | [X]Chronic hallucinatory psychosis | other psychosis |
| Eu2z | [X]Unspecified nonorganic psychosis | other psychosis |
| Eu2z-1 | [X]Psychosis NOS | other psychosis |
| E11-3 | Manic psychoses | other psychosis |
| E1z | Non-organic psychosis NOS | other psychosis |
| 146H | H/O: psychosis | other psychosis |
| E1 | Non-organic psychoses | other psychosis |
| E1y | Other specified non-organic psychoses | other psychosis |
| Eu531-1 | [X]Puerperal psychosis NOS | other psychosis |
| Eu843-4 | [X]Symbiotic psychosis | other psychosis |
| E10 | Schizophrenic disorders | schizophrenia |
| E10-98 | Schizophrenic psychoses NOS | schizophrenia |
| E10-99 | Schizophrenic psychoses | schizophrenia |
| E100 | Simple schizophrenia | schizophrenia |
| E100-1 | Schizophrenia simplex | schizophrenia |
| E1000 | Unspecified schizophrenia | schizophrenia |
| E1001 | Subchronic schizophrenia | schizophrenia |
| E1002 | Chronic schizophrenic | schizophrenia |
| E1003 | Acute exacerbation of subchronic schizophrenia | schizophrenia |
| E1004 | Acute exacerbation of chronic schizophrenia | schizophrenia |
| E1005 | Schizophrenia in remission | schizophrenia |
| E100z | Simple schizophrenia NOS | schizophrenia |
| E101 | Hebephrenic schizophrenia | schizophrenia |
| E1010 | Unspecified hebephrenic schizophrenia | schizophrenia |
| E1011 | Subchronic hebephrenic schizophrenia | schizophrenia |
| E1012 | Chronic hebephrenic schizophrenia | schizophrenia |
| E1013 | Acute exacerbation of subchronic hebephrenic schizophrenia | schizophrenia |
| E1014 | Acute exacerbation of chronic hebephrenic schizophrenia | schizophrenia |
| E1015 | Hebephrenic schizophrenia in remission | schizophrenia |
| E101z | Hebephrenic schizophrenia NOS | schizophrenia |
| E102 | Catatonic schizophrenia | schizophrenia |
| E1020 | Unspecified catatonic schizophrenia | schizophrenia |
| E1021 | Subchronic catatonic schizophrenia | schizophrenia |
| E1022 | Chronic catatonic schizophrenia | schizophrenia |
| E1023 | Acute exacerbation of subchronic catatonic schizophrenia | schizophrenia |
| E1024 | Acute exacerbation of chronic catatonic schizophrenia | schizophrenia |
| E1025 | Catatonic schizophrenia in remission | schizophrenia |
| E102z | Catatonic schizophrenia NOS | schizophrenia |
| E103 | Paranoid schizophrenia | schizophrenia |
| E1030 | Unspecified paranoid schizophrenia | schizophrenia |
| E1031 | Subchronic paranoid schizophrenia | schizophrenia |
| E1032 | Chronic paranoid schizophrenia | schizophrenia |
| E1033 | Acute exacerbation of subchronic paranoid schizophrenia | schizophrenia |
| E1034 | Acute exacerbation of chronic paranoid schizophrenia | schizophrenia |
| E1035 | Paranoid schizophrenia in remission | schizophrenia |
| E103z | Paranoid schizophrenia NOS | schizophrenia |
| E104 | Acute schizophrenic episode | schizophrenia |
| E106 | Residual schizophrenia | schizophrenia |
| E106-1 | Restzustand - schizophrenia | schizophrenia |
| E107 | Schizo-affective schizophrenia | schizophrenia |
| E1070 | Unspecified schizo-affective schizophrenia | schizophrenia |
| E1071 | Subchronic schizo-affective schizophrenia | schizophrenia |
| E1072 | Chronic schizo-affective schizophrenia | schizophrenia |
| E1073 | Acute exacerbation subchronic schizo-affective schizophrenia | schizophrenia |
| E1074 | Acute exacerbation of chronic schizo-affective schizophrenia | schizophrenia |
| E1075 | Schizo-affective schizophrenia in remission | schizophrenia |
| E107z | Schizo-affective schizophrenia NOS | schizophrenia |
| E10y | Other schizophrenia | schizophrenia |
| E10y-1 | Cenesthopathic schizophrenia | schizophrenia |
| E10y0 | Atypical schizophrenia | schizophrenia |
| E10y1 | Coenesthopathic schizophrenia | schizophrenia |
| E10yz | Other schizophrenia NOS | schizophrenia |
| E10z | Schizophrenia NOS | schizophrenia |
| Eu20 | [X]Schizophrenia | bipolar |
| Eu200 | [X]Paranoid schizophrenia | schizophrenia |
| Eu200-1 | [X]Paraphrenic schizophrenia | schizophrenia |
| Eu201 | [X]Hebephrenic schizophrenia | schizophrenia |
| Eu201-1 | [X]Disorganised schizophrenia | schizophrenia |
| Eu202 | [X]Catatonic schizophrenia | schizophrenia |
| Eu202-1 | [X]Catatonic stupor | schizophrenia |
| Eu202-2 | [X]Schizophrenic catalepsy | schizophrenia |
| Eu202-3 | [X]Schizophrenic catatonia | schizophrenia |
| Eu202-4 | [X]Schizophrenic flexibilatis cerea | schizophrenia |
| Eu203 | [X]Undifferentiated schizophrenia | schizophrenia |
| Eu203-1 | [X]Atypical schizophrenia | schizophrenia |
| Eu204 | [X]Post-schizophrenic depression | schizophrenia |
| Eu205 | [X]Residual schizophrenia | schizophrenia |
| Eu205-1 | [X]Chronic undifferentiated schizophrenia | schizophrenia |
| Eu205-2 | [X]Restzustand schizophrenic | schizophrenia |
| Eu206 | [X]Simple schizophrenia | schizophrenia |
| Eu20y | [X]Other schizophrenia | schizophrenia |
| Eu20y-1 | [X]Cenesthopathic schizophrenia | schizophrenia |
| Eu20y-2 | [X]Schizophreniform disord NOS | schizophrenia |
| Eu20y-3 | [X]Schizophrenifrm psychos NOS | schizophrenia |
| Eu20z | [X]Schizophrenia, unspecified | schizophrenia |
| Eu232-4 | [X]Schizophrenic reaction | schizophrenia |
| ZV110 | [V]Personal history of schizophrenia | schizophrenia |
| 1464 | H/O: schizophrenia | schizophrenia |
| **Additional EMIS codes** | **Term** | **Group** |
| EMISQHY1 | Hypomanic | bipolar |
| EGTON118 | Obsessional compulsive psychosis | other psychosis |
| EMISCDE13 | Delusions | other psychosis |
| EMISICD10\|F2381 | Other acute and transient psychotic disorders, with associated acute stress | other psychosis |
| EMISQPA1 | Paranoid | other psychosis |
| EMISICD10\|F2018 | Hebephrenic schizophrenia, other | schizophrenia |
| EMISICD10\|F2035 | Undifferentiated schizophrenia, complete remission | schizophrenia |
| EMISICD10\|F2041 | Post-schizophrenic depression, episodic with progressive deficit | schizophrenia |
| EMISICD10\|F2054 | Residual schizophrenia, incomplete remission | schizophrenia |
| EMISICD10\|F2065 | Simple schizophrenia, complete remission | schizophrenia |
| EMISICD10\|F2098 | Schizophrenia, unspecified, other | schizophrenia |

Supplementary figure 1 - SMI cohort derived from CPRD denominator population

N = 20, 568 SMI

N=2 had SMI and delirium diagnosis on same day

- Outside England
- <1 year follow-up after SMI diagnosis
- Diagnosis <1/4/2000
- Age range
- Eligible for linkage
- Matched at least 1:1

N=39.4 million

Original cohort provided by CPRD

(965,233 ever have a diagnosis of SMI/post-traumatic stress disorder/eating disorder/personality disorder)

N = 235,551 SMI/post-traumatic stress disorder/eating disorder/personality disorder

Exclusions round 1: n=39,164,449

- Sex not recorded
- Mental health diagnosis after end of follow up period
- Mental health diagnosis < 1/1/2000
- <12 months follow up
- Duplicate between CPRD GOLD and Aurum

N = 70,855

SMI defined as schizophrenia, bipolar affective disorder or other non-organic psychosis

n = 164,696

- Eating disorder, PTSD or personality disorder

Exclusions Round 2: n = 2,049

- Under 18 or over 100 at SMI diagnosis
- Record ends before study starts
- Diagnosed before study starts or after study ends
- No region
- Unmatched

N = 68, 806 SMI

Exclusions Round 3: n = 48,238

- Outside England
- <1 year follow-up after SMI diagnosis
- Diagnosis <1/4/2000
- Outside of age range
- Not eligible for linkage
- Unmatched

N = 20, 566 SMI

**SF1: SMI cohort derived from CPRD denominator population.** Patients with SMI were matched up to 1:4 with people without SMI. The same exclusion criteria were applied to matched comparators, though the date of SMI diagnosis of their matched case was used for exclusions based on date of SMI diagnosis. *SMI=severe mental illness, CPRD=Clinical Practice Research Datalink*

Supplementary Table 2 – baseline characteristics by SMI subtype

|  | **No SMI** | **SMI** | **Schizophrenia** | **Bipolar disorder** | **Other psychosis** |
| --- | --- | --- | --- | --- | --- |
| N | 71,374 | 20,566 | 4,553 | 7,454 | 8,559 |
| Year of birth Median (IQR) | 1966 (1950 – 1978) | 1967 (1951 – 1979) | 1969 (1956 – 1979) | 1966 (1953 – 1978) | 1966 (1946 – 1979) |
| Female (%) | 34,758 (48.7) | 10,059 (48.9) | 1,587 (34.9) | 4,354 (58.4) | 4,118 (48.1) |
| Ethnicity (%)  Asian  Black  Mixed  Other  White | 3,924 (5.5)  2,143 (3.0)  514 (0.72)  1,741 (2.44)  63,052 (88.3) | 1,077 (5.24)  998 (4.85)  237 (1.15)  507 (2.47)  17,747 (86.3) | 367 (8.06)  418 (9.18)  66 (1.45)  118 (2.59)  3,584 (78.7) | 248 (3.33)  162 (2.17)  68 (0.91)  196 (2.63)  6,780 (91.0) | 462 (5.4)  418 (4.88)  103 (1.20)  193 (2.25)  7,383 (86.3) |
| Region (%)  East Midlands  East of England  London  North East  North West  South Central  South EC  South West  West Midlands  Y&TH | 2,415 (3.38)  6,255 (8.76)  13,849 (19.4)  1,471 (2.06)  9,465 (13.3)  9,812 (13.8)  6,861 (9.61)  9,987 (14.0)  8,502 (11.9)  2,757 (3.86) | 664 (3.23)  1,740 (8.46)  4,161 (20.2)  407 (1.98)  2,720 (13.2)  2,837 (13.8)  1,996 (9.71)  2,892 (14.1)  2,389 (11.6)  760 (3.70) | 135 (2.97)  307 (6.74)  1,286 (28.3)  78 (1.71)  645 (14.2) 528 (11.6)  367 (8.06)  490 (10.8)  564 (12.4)  153 (3.36) | 234 (3.14)  703 (9.43)  1,203 (16.1)  153 (2.05)  882 (11.8)  1,184 (15.9)  965 (12.9)  1,112 (14.9)  771 (10.3)  247 (3.31) | 295 (3.45)  730 (8.53)  1,672 (19.5)  176 (2.06)  1,193 (13.9)  1,125 (13.1)  664 (7.76)  1,290 (15.1)  1,054 (12.3)  360 (4.21) |
| IMD quintile (%)  1 – least deprived  2  3  4  5 – most deprived | 13,637 (19.1)  13,891 (19.4)  13,970 (19.6)  15,674 (22.0)  14,202 (19.9) | 3,057 (14.9)  3,3337 (16.2)  3,876 (18.8)  4,847 (23.6)  5,450 (26.5) | 435 (9.55)  537 (11.8)  812 (17.8)  1,182 (26.0)  1,587 (34.9) | 1,440 (19.3)  1,453 (19.5)  1,462 (19.6)  1,606 (21.6) 1,493 (20.0) | 1,181 (13.8)  1,347 (15.7)  1,602 (18.7)  2,059 (24.1)  2,370 (27.7) |
| Physical comorbidities at index (%)  None  One  More than one | 43,478 (60.9)  17,082 (23.9)  10,814 (15.2) | 11,579 (56.3)  5,308 (25.8)  3,679 (17.9) | 3,009 (66.1)  1,045 (22.9)  499 (11.0) | 4,117 (55.2)  2,033 (27.3)  1,304 (17.5) | 4,453 (52.0)  2,230 (26.1)  1,876 (21.9) |

**ST2: Baseline characteristics by SMI subtype.** Continuous variables are displayed as median (IQR). Categorical variables are displayed as n(%),to 3 significant figures. *SMI=severe mental illness, IQR=interquartile range, IMD=index of multiple deprivation*

Supplementary Table 3 - ICD-10 codes used to identify delirium

| **ICD-10 code** | **Number of diagnoses identified** |
| --- | --- |
| **F05 Delirium, not induced by alcohol and other psychoactive substances** |  |
| F05.0 Delirium, not superimposed on dementia | 65 |
| F05.1 Delirium, superimposed on dementia | 147 |
| F05.8 Other delirium | 58 |
| F05.9 Delirium, unspecified | 1,016 |
| **G Acute encephalopathy** |  |
| G04.30 Acute necrotising hemorrhagic encephalopathy | 0 |
| G04.31 Post infectious acute necrotising hemorrhagic encephalopathy | 0 |
| G04.32 Post immunization acute necrotising hemorrhagic encephalopathy | 0 |
| G92 Toxic encephalopathy | 3 |
| G93.4 Acute encephalopathy, unspecified | 94 |
| G93.41 Metabolic encephalopathy | 0 |
| G93.49 Other encephalopathy | 0 |
| **Total:** | 1,383* |

*1,375 episodes with 1 delirium diagnosis, 4 episodes with 2 different delirium diagnoses

Supplementary Table 4 - Assessment for linkage bias

|  | **Not eligible for linkage** | **Eligible for linkage** |
| --- | --- | --- |
| n (%) | 144,853 (61.07) | 92,320 (38.93) |
| **Data source (%)** CPRD Gold (%) | 13,052 (9.01) | 43,583 (47.21) |
| CPRD Aurum (%) | 131,801 (91.00) | 48,737 (52.79) |
| **SMI diagnosis (%)** |  |  |
| None | 113,269 (78.20) | 71,674 (77.64) |
| Schizophrenia | 7,314 (5.05) | 4,581 (4.96) |
| Bipolar | 11,385 (7.86) | 7,480 (8.10) |
| Other | 12,885 (8.90) | 8,585 (9.30) |
| **Age at index (median [IQR])** | 41.63 [30.13, 56.46] | 41.62 [29.72, 56.94] |
| **Female (%)** | 71478 (49.35) | 45,015 (48.76) |
| **Region (%)** | |  |
| East Midlands | 2,732 (1.89) | 3,084 (3.34) |
| East of England | 7,206 (4.97) | 8,015 (8.68) |
| London | 29,060 (20.06) | 18,086 (19.59) |
| North East | 8,415 (5.81) | 1,878 (2.03) |
| North West | 31,046 (21.43) | 12,219 (13.24) |
| South Central | 12,735 (8.79) | 12,663 (13.72) |
| South East Coast | 10,013 (6.91) | 9,008 (9.76) |
| South West | 13,899 (9.60) | 12,929 (14.00) |
| West Midlands | 23,832 (16.45) | 10,921 (11.83) |
| Yorkshire And The Humber | 5,915 (4.08) | 3,517 (3.81) |
| **Patient-level IMD (%)** | |  |
| 1 – least deprived | 2 (0.00) | 16,830 (18.23) |
| 2 | 3 (0.00) | 17,265 (18.70) |
| 3 | 2 (0.00) | 17,886 (19.37) |
| 4 | 3 (0.00) | 20,537 (22.25) |
| 5 – most deprived | 1 (0.00) | 19,655 (21.29) |
| Missing | 144,842 (99.99) | 147 (0.16) |
| **Ethnicity (%)** | |  |
| Asian | 7,258 (5.01) | 5,016 (5.43) |
| Black | 5,816 (4.02) | 3,157 (3.42) |
| Mixed | 1,552 (1.07) | 755 (0.82) |
| Other | 2,500 (1.73) | 2,259 (2.45) |
| White | 75,270 (51.96) | 42,163 (45.67) |
| Missing | 52,457 (36.21) | 38,970 (42.21) |
| **Comorbidities (%)** |  |  |
| No comorbidities | 67,754 (46.77) | 43,223 (46.82) |
| One comorbidity | 35,276 (24.35) | 22,929 (24.84) |
| More than one comorbidity | 41,823 (28.87) | 26,168 (28.34) |
| **Deaths (%)** | 10,587 (7.31) | 6,648 (7.20) |
| **Age at death (median [IQR])** | 80.91 [67.96, 88.27] | 82.11 [69.76, 89.12] |
| **Follow-up time (median [IQR])** | 4.90 [2.53, 9.05] | 4.74 [2.49, 8.50] |

**ST4: Assessment for linkage bias.** Continuous variables are displayed as median (IQR). Categorical variables are displayed as n(%),to 3 significant figures. *Age at index date is age on date of SMI diagnosis, and for matched comparators is age on the date their matched SMI case was diagnosed. SMI=severe mental illness, IQR=interquartile range, IMD=index of multiple deprivation

Supplementary figure 2 – testing for proportional hazards

When comparing the hazard rate of delirium in patients with SMI to matched comparators without SMI, in the younger group (18-64 years), Schoenfeld tests showed non-proportionality in the unadjusted analysis (Fig 2a, p=0.0066). However when adjusting for the covariates in the model; age, sex, ethnicity, deprivation and calendar year, Schoenfeld residuals showed proportionality (Fig 2b, p=0.9361). Similarly, in the older group (≥65 years), Schoenfeld tests showed non-proportionality in the unadjusted analysis (Fig 2c, p=0.0001), but proportionality in the adjusted analysis (Fig 2d, p=0.9076).


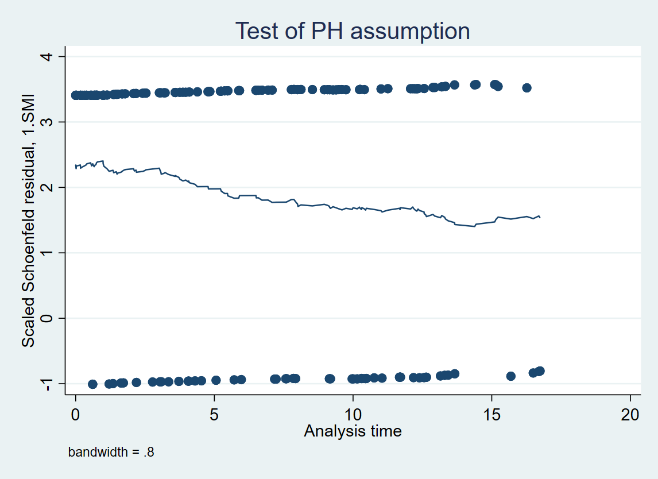

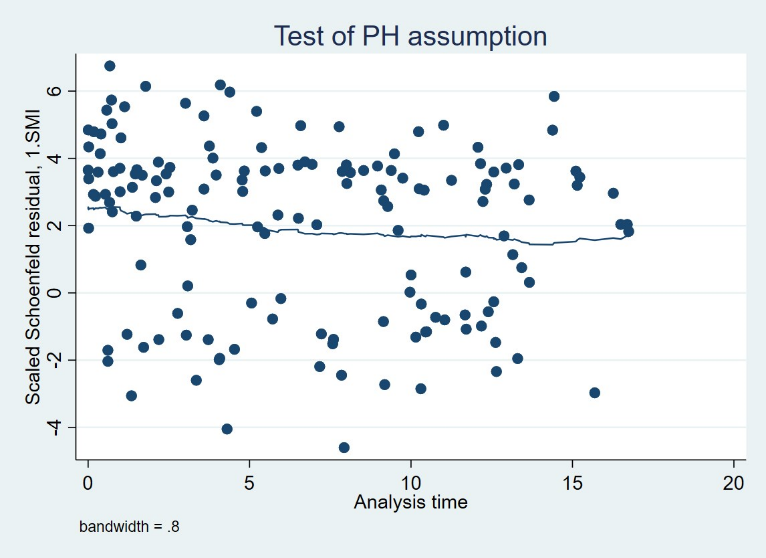

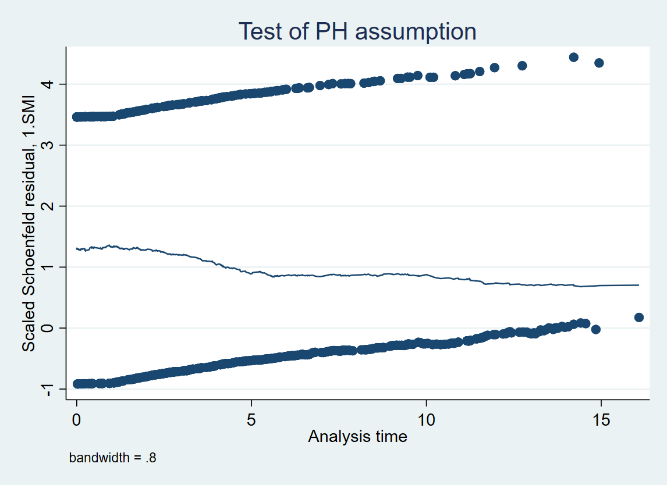

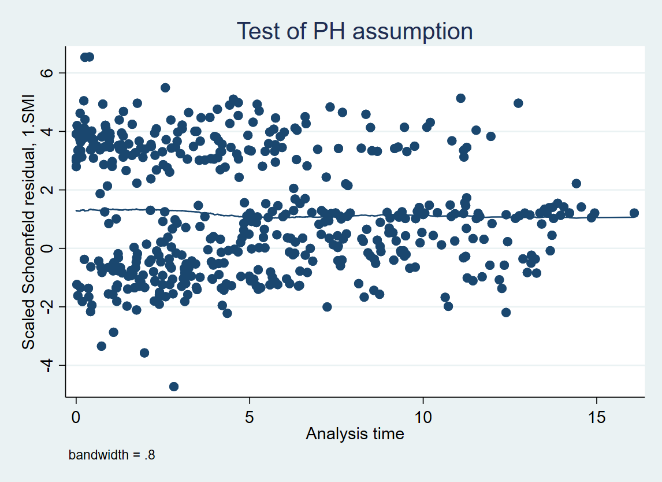


**A**

**B**

**C**

**D**

**SF2: Schoenfeld residual plots.** Figures A-D show plots of Schoenfeld residuals for SMI *vs.* non-SMI with respect to recorded hospital delirium diagnoses. A=18-64 year group, unadjusted model. B=18-64 year group, adjusted for age, calendar year, sex, ethnicity and deprivation level. C=≥65 years, unadjusted model. D=≥65 years, adjusted for age, calendar year, sex, ethnicity and deprivation level. *SMI=severe mental illness, PH=proportional hazards*

Supplementary figure 3 – Kaplan-Meier survival curves

**A**

**B**

**C**

**D**

**SF3: Kaplan-Meier survival curves for probability of remaining delirium-free during follow-up.** A=younger age group (18-64 years) by SMI status, B=older age group (≥65 years) by SMI status, C=younger age group (18-64 years) by SMI subtype, D=older age group (≥65 years) by SMI subtype. X-axis displays years since SMI diagnosis or since equivalent date for matched non-SMI comparators. *SMI=severe mental illness*

Supplementary table 5 – sensitivity analysis

|  |  | ***No washout*** | | ***3-month washout*** | | ***6-month washout*** | |
| --- | --- | --- | --- | --- | --- | --- | --- |
|  | **Age group** | **N** | **aHR (95% CI)** | **N** | **aHR (95% CI)** | **N** | **aHR (95% CI)** |
| **No SMI** | 18-64y | 59,208 | 1 (ref) | 59,176 | 1 (ref) | 59,165 | 1 (ref) |
| **SMI** |  | 17,092 | 7.65  (5.45 –10.7) | 17,084 | 6.97  (4.94 – 9.84) | 17,081 | 6.72  (4.75 – 9.50) |
| **No SMI** | ≥65y | 12,166 | 1 (ref) | 12,099 | 1 (ref) | 12,057 | 1 (ref) |
| **SMI** |  | 3,474 | 3.35  (2.77 – 4.05) | 3,456 | 3.03  (2.49 – 3.68) | 3,444 | 2.81  (2.30 – 3.43) |
| **Total n** | | 91,940 | | 91,815 | | 91,747 | |

**ST5: Hazard ratios for hospital delirium diagnosis by SMI status with 3 and 6-month washout periods applied.** Comparators matched to the patients falling within the washout periods were also excluded. Total N for each analysis is displayed. Hazard ratios are reported to 2 decimal places. All p values were <0.001. *SMI=severe mental illness, HR=hazard ratio, CI=Confidence interval, ref=reference category for hazard ratios*

Supplementary table 6 – characteristics of subset who died within 30 days of hospitalisation involving delirium diagnosis compared to delirium subset and full cohort

|  | **Full cohort** | **Delirium (after 1 year of follow-up)** | **Died within 30 days of delirium hospitalisation** |
| --- | --- | --- | --- |
| N | 91,940 | 554 | 85 |
| SMI diagnosis  Schizophrenia  Bipolar disorder  Other Psychosis  None | 20,566 (22.4)  4,553 (4.95)  7,454 (8.11)  8,559 (9.30)  71,374 (77.6) | 210 (37.9)  33 (5.96)  79 (14.3)  98 (17.7)  344 (62.1) | 24 (28.2)  4 (4.71)  14 (16.5)  6 (7.06)  61 (71.8) |
| Follow-up time (years) (IQR) | 4.74 (2.49 – 8.50) | 7.11 (4.10 – 11.0) | 6.78 (3.95 – 10.1) |
| Year of birth Median (IQR) | 1966 (1950 – 1978) | 1931 (1924 – 1940) | 1930 (1924 – 1937) |
| Age at index (IQR) | 41.6 (29.7 – 56.9) | 76.5 (66.4 – 83.7) | 76.6 (68.8 – 84.2) |
| Female (%) | 44,817 (48.8) | 360 (65.0) | 41(48.2) |
| Ethnicity (%)  Asian  Black  Mixed  Other  White | 5,001 (5.44)  3,141 (3.42)  751 (0.82)  2,248 (2.45)  80,799 (87.9) | 10 (1.81)  16 (2.89)  4 (0.72)  16 (2.89)  508 (91.7) | 3 (3.53)  0 (0)  0 (0)  0 (0)  82 (96.5) |
| Region (%)  East Midlands  East of England  London  North East  North West  South Central  South EC  South West  West Midlands  Y&TH | 3,079 (3.35)  7,995 (8.70)  18,010 (19.6)  1,878 (2.04)  12,185 (13.3)  12,649 (13.8)  8.857 (9.63)  12,879 (14.0)  10,891 (11.9)  3,517 (3.83) | 12 (2.17)  34 (6.14)  105 (19.0)  20 (3.61)  104 (18.8)  58 (10.5)  57 (10.3)  84 (15.2)  65 (11.7)  15 (2.71) | 3 (3.53)  7 (8.24)  12 (14.1)  6 (7.06)  15 (17.7)  7 (8.24)  9 (10.6)  10 (11.8)  14 (16.5)  2 (2.35) |
| IMD quintile (%)  1 – least deprived  2  3  4  5 – most deprived | 16,693 (18.2)  17,228 (18.7)  17,846 (19.4)  20,521 (22.3)  19,652 (21.4) | 94 (17.0)  91 (16.4)  116 (20.9)  113 (20.4)  140 (25.3) | 16 (18.8)  11 (12.9)  16 (18.8)  17 (20.0)  25 (29.4) |
| Physical comorbidities at index (%)  None  One  More than one | 55,057 (59.9)  22,380 (24.4)  14,493 (15.8) | 126 (22.7)  119 (21.5)  309 (55.8) | 19 (22.4)  18 (21.2)  48 (56.5) |

**ST6: Characteristics of those who died within 30 days of hospitalisation involving delirium diagnosis compared to full cohort and delirium subset.** Continuous variables are displayed as median (IQR). Categorical variables are displayed as n(%), reported to 3 significant figures). *SMI=severe mental illness, IQR=interquartile range, IMD=Index of multiple deprivation*

Supplementary Table 7 – Hazard ratios for incidence of delirium diagnosis by gender, ethnicity and deprivation level

| Age group |  | Hazard ratio (95% CI) | p-value |
| --- | --- | --- | --- |
| 18-64 years | **SMI**  1  0 | 1 (ref)  7.65 (5.45 – 10.7) | <0.001 |
|  | **Gender**  Male  Female | 1 (ref)  0.65 (0.45 – 0.94) | 0.022 |
|  | **Ethnicity**  White  Asian  Black  Mixed  Other | 1 (ref)  0.49 (0.19 – 1.25)  0.84 (0.25 – 2.77)  4.91 (1.51 – 16.0)  9.87 (0.25 – 2.99) | 0.133  0.769  0.008  0.829 |
|  | **IMD**  1 (least deprived)  2  3  4  5 (most deprived) | 1 (ref)  0.77 (0.32 – 1.86)  3.76 (1.89 – 7.47)  2.54 (1.26 – 5.11)  2.98 (1.45 – 6.12) | 0.566  <0.001  0.009  0.003 |
| ≥65 years | **SMI**  1  0 | 1 (ref)  3.35 (2.77 – 4.05) | <0.001 |
|  | **Gender**  Male  Female | 1 (ref)  0.84 (0.65 – 1.08) | 0.173 |
|  | **Ethnicity**  White  Asian  Black  Mixed  Other | 1 (ref)  0.99 (0.49 – 2.01)  1.12 (0.63 – 2.01)  2.33 (0.92 – 5.95)  1.39 (0.70 – 2.76) | 0.990  0.703  0.076  0.345 |
|  | **IMD**  1 (least deprived)  2  3  4  5 (most deprived) | 1 (ref)  1.41 (1.03 – 1.94)  1.36 (0.98 – 1.88)  1.28 (0.91 – 1.82)  1.58 (1.08 – 2.30) | 0.030  0.063  0.161  0.017 |

**ST7: Hazard ratios for incidence of delirium diagnosis by gender, ethnicity and deprivation level.** Hazard ratios and confidence intervals reported to 2 decimal places. *SMI=severe mental illness, IQR=interquartile range, IMD=Index of multiple deprivation*
